# Responding to the Unique Complexities of Fetal Alcohol Spectrum Disorder

**DOI:** 10.3389/fpsyg.2021.778471

**Published:** 2022-01-25

**Authors:** Katherine Flannigan, Jacqueline Pei, Kaitlyn McLachlan, Kelly Harding, Mansfield Mela, Jocelynn Cook, Dorothy Badry, Audrey McFarlane

**Affiliations:** ^1^Canada Fetal Alcohol Spectrum Disorder Research Network, Vancouver, BC, Canada; ^2^Department of Educational Psychology, University of Alberta, Edmonton, AB, Canada; ^3^Department of Psychology, University of Guelph, Guelph, ON, Canada; ^4^Department of Psychology, Laurentian University, Sudbury, ON, Canada; ^5^Department of Psychiatry, University of Saskatchewan, Saskatoon, SK, Canada; ^6^Society of Obstetricians and Gynaecologists of Canada, Ottawa, ON, Canada; ^7^Department of Obstetrics and Gynecology, University of Ottawa, Ottawa, ON, Canada; ^8^Faculty of Social Work, University of Calgary, Calgary, AB, Canada

**Keywords:** fetal alcohol spectrum disorder (FASD), neurodevelopmental disability, complex needs, research advances, practice and policy issues

## Abstract

Fetal alcohol spectrum disorder (FASD) is a multifaceted disability, characterized not only by brain- and body-based challenges, but also high rates of environmental adversity, lifelong difficulties with daily living, and distinct sociocultural considerations. FASD is one of the most common neurodevelopmental disabilities in the Western world and associated with significant social and economic costs. It is important to understand the complexities of FASD and the ways in which FASD requires unique consideration in research, practice, and policy. In this article, we discuss our perspectives on factors that distinguish FASD from other disabilities in terms of complexity, co-occurrence, and magnitude. We provide an overview of select literature related to FASD as a socially rooted disability with intergenerational impacts and multiple layers of stigma. These social issues are intertwined with notable experiences of adversity across the lifespan and high rates of co-occurring health concerns for individuals with FASD, all of which present unique challenges for individuals, caregivers, families, service providers, and policy makers. Understanding these factors is the first step in developing and implementing specialized initiatives in support of positive outcomes for individuals with FASD and their families. Future directions are proposed for advancing research, practice, and policy, and responding to the unique complexities of FASD.

## Introduction

At least 4–5% of individuals in Canada and the United States are estimated to have fetal alcohol spectrum disorder [Fetal alcohol spectrum disorder (FASD); May et al., 2018; Popova et al., 2019]. Although it is one of the most common neurodevelopmental disabilities (NDDs) in the Western world, knowledge and awareness of FASD within the public and among service providers continues to lag compared with other disabilities (Johnson et al., 2010; Mukherjee et al., 2015b; Choate et al., 2019). FASD stems from prenatal alcohol exposure (PAE) and is characterized by cognitive, behavioral, emotional, social, and adaptive difficulties (Cook et al., 2016) along with many co-occurring physical and mental health concerns (Popova et al., 2016; Himmelreich et al., 2020). Without adequate support, individuals with FASD can experience a range of negative outcomes, but positive trajectories may be encouraged through early diagnosis, intervention, and high-quality caregiving environments (Streissguth et al., 2004).

## Complexities of Fetal Alcohol Spectrum Disorder

**FIGURE 1:**
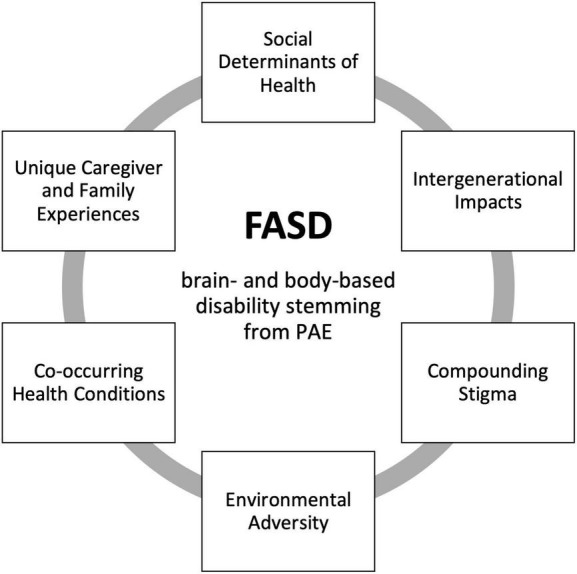
Factors contributing to the unique complexity of FASD. FASD, fetal alcohol spectrum disorder; PAE, prenatal alcohol exposure.

Fetal alcohol spectrum disorder is a unique NDD (Di Pietro and Illes, 2016), distinct from other disabilities through a combination of several inter-related and compounding factors (see Figure 1). Although these factors may be relevant across disability groups, FASD is distinct in terms of the complexity, co-occurrence, and magnitude with which they occur. It is critical for researchers, service providers, and policy makers to understand factors that contribute to the complexity of FASD. With increased awareness and understanding comes the potential to improve resources, strengthen advocacy efforts, and ultimately promote wellbeing and positive outcomes for individuals with FASD, their families, and communities. Here, we present our perspectives on the unique complexities of FASD, informed by our collective experience and expertise in clinical psychology, psychiatry, women’s health, disability, child welfare, and social policy, situated within the context of the current evidence base.

### Prenatal Alcohol Exposure and Social Determinants of Health

There are myriad factors that both directly and indirectly influence why a pregnant woman may consume alcohol, many of which parallel the social determinants of health (SDHs; Government of Canada, 2020). Experiences of trauma (Bhengu et al., 2019); stressful life events (Edwards et al., 2019); intimate partner violence (Deutsch, 2019); having a partner who uses substances (Ortega-García et al., 2020); lower levels of education (May et al., 2020); less or later access of prenatal health care (May et al., 2020; Popova et al., 2021); and mental health challenges (Hyer et al., 2020; Popova et al., 2021) have all been associated with an increased risk of alcohol use during pregnancy. Many pregnancies are unplanned, and not all women recognize their pregnancy during the early stages. People may also have limited awareness about the harms associated with alcohol use during pregnancy (Bitew et al., 2020), or may have received conflicting information from the media or health care professionals (Elek et al., 2013). Although other disabilities have been associated with SDHs in that SDHs can be influenced by disability status (Frier et al., 2018), or may impact caregiver beliefs (Zuckerman et al., 2015) and wellbeing (Spencer et al., 2021), with FASD, compromised SDHs are uniquely associated as potentially causal factors.

Given the association between prenatal alcohol exposure (PAE) and SDHs (Abadir and Ickowicz, 2016), comprehensive initiatives are needed to reduce the complex risk factors linked to alcohol use during pregnancy and promote health and wellness in a way that is respectful and responsive to high levels of vulnerability (Poole et al., 2016; Hubberstey et al., 2019).

### Intergenerational Impacts

Researchers have recently begun to explore the multi-generational contributors to FASD. Although the specific mechanisms underlying the intergenerational aspects of FASD are not fully understood, possible genetic and epigenetic contributors have been examined (Govorko et al., 2012; Harper et al., 2014; Mead and Sarkar, 2014; Popoola et al., 2017). PAE is an early life stressor that can damage the developing brain and cause neurological alterations leading to a heightened vulnerability to stress, mental health problems, and substance use (Weinberg et al., 2008; Hellemans et al., 2010; Ciafrè et al., 2020). In animal models of PAE, researchers have found that the brain’s altered stress response system and resulting epigenetic impacts can be carried on for at least three generations (Nizhnikov et al., 2016). Environmental stressors and experiences of trauma may further exacerbate these risk factors. In one study, grandmothers of children with FASD were more likely to have experienced trauma, injuries, and difficulties related to alcohol use compared to grandmothers of children without FASD (Kvigne et al., 2008). Other researchers have reported that individuals with FASD experience high rates of intergenerational trauma (Samaroden, 2018), alcohol and substance use (Dodge et al., 2019; Goldschmidt et al., 2019), as well as risky sexual behavior (De Genna et al., 2015). When these behaviors and experiences are combined, in the absence of adequate services and supports, individuals with FASD may themselves be at increased risk of having future alcohol-exposed pregnancies.

The causal pathways of FASD are multi-faceted and complex (McQuire et al., 2020), involving genetic and biological, behavioral, interpersonal, sociocultural, and historical factors. Together, these pathways can increase the risk of trauma, health and economic disparity, substance use, and other risk factors that perpetuate the intergenerational cycle of FASD (Meurk et al., 2014; Cloete and Ramugondo, 2015; Gonzales et al., 2021).

### Compounding Stigma

Research on stigma and NDDs is relatively scarce; however, there is some evidence that FASD is stigmatized in numerous compounding ways. Like other disabilities, stigma affects individuals with FASD and their care providers through experiences of marginalization, negative stereotypes, lower self-esteem, and misperceptions about the individual’s abilities (Bell et al., 2015). Negative attitudes about life trajectories are prevalent, with the positive potential of individuals with FASD overshadowed by a public perception that negative outcomes are inevitable (Bell et al., 2015; Olson and Sparrow, 2021). This stigma can create significant barriers to service access for individuals with FASD and their caregivers by undermining their willingness to seek supports (Bell et al., 2015). Moreover, these barriers may be compounded by a misunderstanding of FASD among professionals, and the disqualification of individuals with FASD from mainstream services (Anderson et al., 2019). Even service providers who are knowledgeable about the disability may hesitate to diagnose an individual with FASD because of concerns about how stigma will impact the individual and their family (Payne et al., 2005; Elliott et al., 2006; Mukherjee et al., 2015a; Howlett et al., 2019).

In addition to the general stigmatization that impacts people across disabilities groups, FASD is uniquely seen through a “criminalized” public lens (Bell et al., 2015; Aspler et al., 2018). In the media, individuals with FASD are often portrayed as people who commit crimes, which perpetuates harmful generalizations about criminality and further stigmatizes individuals with FASD (Aspler et al., 2018). Another layer of stigma that is unique to FASD is the shame and blame that is targeted toward women and mothers (Corrigan et al., 2017). FASD has been inextricably linked to women’s behavior and mothers are often held responsible for “victimizing” (Aspler et al., 2019) or “causing” harm to their child (Bell et al., 2015). This stigma can be particularly harmful as it impedes women from seeking support or discussing alcohol consumption with care providers out of fear of judgment, child removal, or incarceration (Poole et al., 2016). Because the direct cause of FASD is PAE, it may be seen as preventable, in principle. However, this oversimplification can be misleading and harmful, further stigmatizing and marginalizing women who consume alcohol during pregnancy, as well as individuals with FASD and their families (Roozen et al., 2020).

Another unique layer of stigma in FASD exists at the sociocultural level, whereby it is commonly, and incorrectly, misperceived to be an “Indigenous issue” (McKenzie et al., 2016; Aspler et al., 2019). In fact, there are no recent or rigorous prevalence studies to support this misconception (Flannigan et al., 2018). Although cultural factors are relevant to FASD insofar as they can help to guide prevention, diagnosis, and intervention efforts (Samaroden, 2018; Pei et al., 2019; Gibson et al., 2020; Hamilton et al., 2020; Gonzales et al., 2021), cultural stigma can cause substantial harm. By disproportionately focusing on FASD in Indigenous communities, not only are negative stereotypes perpetuated, but individuals with other NDDs may be misdiagnosed or overlooked, which can impact resource allocation and lead to a lack of appropriate supports (Di Pietro and Illes, 2014, 2016). There is a critical need to address the harms of cultural stigma and support FASD work in Indigenous communities through community-led approaches with consideration of contextual factors, historical and contemporary trauma, and the ongoing marginalization of Indigenous peoples and communities (Gonzales et al., 2021).

Multi-layered and targeted efforts are needed to reduce the stigma associated with FASD at the individual, familial, community, and societal levels to better understand, respect, and provide services for individuals with FASD and their families.

### Environmental Adversity

Individuals with FASD experience disproportionately high rates of prenatal and postnatal adversity, which has profound impacts on their developmental trajectories (Price et al., 2017; Lebel et al., 2019). Beginning early in life, exposure to adverse experiences such as caregiving disruption, death of a parent, abuse and neglect, and exposure to familial substance use, violence, mental health problems, and criminal justice system involvement, are well-documented in this population (Coggins et al., 2007; Koponen et al., 2009; McLachlan et al., 2015, 2016; Kambeitz et al., 2019). Adverse experiences continue throughout the lifespan for individuals with FASD, with high rates of problems with school, employment, housing, independence, victimization, and legal involvement (Streissguth et al., 2004; Rangmar et al., 2015; McLachlan et al., 2020). Although environmental adversity is common for individuals across disability groups (Austin et al., 2016), including those with developmental disabilities (Emerson, 2014; Reichman et al., 2018), it is especially pervasive, chronic, and complex among those with FASD (McLachlan et al., 2015, 2020; Kambeitz et al., 2019; Flannigan et al., 2021a).

Exposure to adversity, particularly in the early years, can have a profoundly damaging and cumulative effect on an individual’s long-term health and wellbeing (Felitti et al., 1998; Chartier et al., 2010). Environmental adversity can exacerbate the brain-based vulnerability resulting from PAE and further increase the risk of negative outcomes (Price et al., 2017).

Considering the increased biopsychosocial vulnerability of individuals with FASD, supports and services should be delivered early, and incorporate a holistic, family-focused, and long-term approach to promoting safety, stability, and wellbeing.

### Co-occurring Conditions

Although co-occurring mental health issues are common for individuals with various developmental disabilities (Cooper et al., 2007; Simonoff et al., 2008), the rates of comorbidity tend to be higher among those with FASD. It is estimated that 90% or more of individuals with FASD experience co-occurring neurodevelopmental and mental health diagnoses (Streissguth et al., 2004; Pei et al., 2011; Weyrauch et al., 2017; Temple et al., 2019). Compared to the general population, individuals with FASD are reported to be 10 times more likely to have ADHD, 20 times more likely to have substance use problems, and 25 times more likely to be diagnosed with a psychotic disorder (Popova et al., 2016; Weyrauch et al., 2017). Challenges with substance use (Dodge et al., 2019; Goldschmidt et al., 2019) and suicidality (Dirks et al., 2019; O’Connor et al., 2019; Flannigan et al., 2021b) are also common in this population.

Mental health challenges among individuals with FASD occur alongside complex biopsychosocial vulnerabilities, which may obstruct a clear understanding of the underlying needs of the individual. FASD is a heterogeneous disability and the varying ways in which FASD and comorbid conditions can manifest and are interpreted have serious implications for diagnosis and treatment (Grant et al., 2013). The presence of multiple health conditions can make it difficult for service providers to identify the root cause of symptoms or behaviors, assign an accurate diagnosis, and apply effective interventions. Moreover, most individuals with FASD show no obvious physical signs of impairment (Andrew, 2011), and the “hidden” nature of the disability can further compound the challenges of accurate identification. There is a critical lack of mental health and substance use interventions for individuals with FASD (Flannigan et al., 2020). Furthermore, a lack of recognition of the potential for wellness and resilience among individuals with FASD may create barriers to service access where mental health and substance use treatments are denied because of service providers’ misperceptions about the disability (Anderson et al., 2019).

The clinical complexities of FASD underscore the multifaceted needs of individuals with this disability and highlight the importance of effective communication, professional education strategies, as well as comprehensive and needs-driven services and supports (Pei et al., 2021).

### Caregiver and Family Experiences

The complex needs associated with FASD present significant challenges for caregivers, and can result in distress, isolation, grief, and loss (Paley et al., 2006; Sanders and Buck, 2010; Kautz et al., 2020; Skorka et al., 2020). Caregivers often report feeling under-supported, misunderstood, and at times, blamed by service providers for the challenges of their family member with FASD (Mukherjee et al., 2013; Coons et al., 2018; Domeij et al., 2018). Although these concerns may be a reality for families of individuals with any disability (Heifetz et al., 2019; Miranda et al., 2019; Marquis et al., 2020; Masefield et al., 2020), caregivers of those with FASD experience stressors that are unique, and in some cases more severe, relative to other disability populations. For instance, there is emerging evidence suggesting that parents of children with FASD experience significantly higher levels of pessimism, poor parent-child interactions, difficult child characteristics, and less hope for the future, compared to parents of children with autism (Watson et al., 2013a,b). Importantly, because of limited service availability for individuals with FASD as they transition to adulthood (Burnside and Fuchs, 2013), many caregivers will play a long-standing role in assisting and advocating for their family member in navigating support systems. Notably, caregivers also demonstrate remarkable adaptability in raising individuals with FASD (Coons et al., 2016).

Caregiver challenges become more complicated when situated in the broader context of stigmatization, intergenerational impacts, and chronic adversity associated with FASD. As such, evidence-based interventions to support caregivers of individuals with FASD are essential (Mohamed et al., 2020). Comprehensive networks of support that leverage caregiver strengths and community resources should be prioritized.

## Discussion and Future Directions

A growing body of evidence highlights the unique complexity of FASD and informs our understanding of the diverse factors that must be addressed to support wellbeing and positive trajectories for this population. This research lays a foundation for the advancement of FASD research, practice, and policy.

There is an ongoing need to address the individual and biopsychosocial factors that contribute to alcohol use during pregnancy through targeted supports for people who experience these risk factors. Initiatives are needed to improve public awareness of FASD and to reduce the stigma, shame, and blame experienced by individuals with FASD, their parents, families, and the broader community. Resources are needed to better support women’s overall health and wellbeing in order to reduce the likelihood of PAE, and ultimately improve outcomes for women, children, and families (Hubberstey et al., 2019). Given the intergenerational impacts of FASD, practice and policy initiatives should address the broader social and systemic inequities that place multiple generations of families at risk for FASD. To be as comprehensive and effective as possible, FASD interventions should contextualize the needs and challenges of the individual within their larger family system.

Supports for caregivers and families are urgently needed (Bobbitt et al., 2016), especially those caring for transition-aged youth and adults with FASD who experience needs that are highly complex (McLachlan et al., 2020). Caregiver supports should emphasize self-care, provide support for grief and loss, offer respite, promote social connection, enhance advocacy, and consider the multi-generational impacts of FASD. The responsibility of supporting an individual with FASD across the lifespan should not fall solely on caregivers, and community-based supports should be enhanced to include natural networks that meaningfully foster interdependence for individuals with FASD and their families.

Targeted efforts are needed to increase knowledge of FASD among service providers, improve access for individuals with FASD to needed programming, and ensure that services account for the unique brain-based differences associated with PAE. Service providers should receive specific training on the complexities of FASD to effectively identify and support clients with FASD. Training should involve a prevention component and emphasize compassionate approaches to facilitating safe discussions about alcohol use. Improved knowledge and sensitivity to alcohol use during pregnancy will help to ensure that women and families are adequately supported in seeking services. FASD curricula at the postsecondary level would be especially useful to ensure that professionals are equipped at the outset of their careers with knowledge and strategies specific to FASD.

Funding and resources are needed to develop and implement FASD-specific supports for individuals across the lifespan. FASD services should be individualized, interdisciplinary, trauma-informed, culturally appropriate, family-centered, and lifelong to ensure comprehensive systems of support. Moreover, services should be integrated across systems to address the diverse needs of this population (Pei et al., 2021). More work is needed to explore the ways in which FASD is distinct from other disabilities, as well as shared needs between groups to inform the development of specialized supports. FASD-specific research, practice, and policy must draw upon the lived experiences of individuals with FASD and their families to ensure that their perspectives and realities are meaningfully considered.

Finally, much of the current literature is focused on the challenges associated with FASD, with a critical gap in terms of strengths and successful outcomes (Olson and Sparrow, 2021). Strengths-based work is critical for reducing the stigma associated with FASD, and for identifying and leveraging the positive potential of individuals with FASD and their care providers. Despite the complexities of the disability, individuals with FASD can thrive, and there is an urgent need to provide opportunities for them to do so.

## Conclusion

Fetal alcohol spectrum disorder represents the intersection of complicated biological, family, community, and societal circumstances that increase risk for social inequity, intergenerational trauma, and health disparity. To fully understand FASD and its associated challenges, and to effectively identify and support individuals with FASD and their families, it is necessary to contextualize the disability within this complex web of risk and vulnerability. Working with individuals with FASD and their families requires empathy, flexibility, creativity, resourcefulness, and cross-disciplinary collaboration. FASD is a significant social and health issue, and targeted work is needed to better address the unique challenges associated with the disability, recognize and build strengths and resilience, and promote the long-term wellbeing of individuals with FASD, their families, and their communities.

## Data Availability Statement

The original contributions presented in the study are included in the article/supplementary material, further inquiries can be directed to the corresponding author.

## Author Contributions

KF wrote the first draft. JP, KM, KH, and MM contributed writing to sections of the manuscript. All authors contributed to the conception of this manuscript and read, revised, and approved the submitted version.

## Conflict of Interest

The authors declare that the research was conducted in the absence of any commercial or financial relationships that could be construed as a potential conflict of interest.

## Publisher’s Note

All claims expressed in this article are solely those of the authors and do not necessarily represent those of their affiliated organizations, or those of the publisher, the editors and the reviewers. Any product that may be evaluated in this article, or claim that may be made by its manufacturer, is not guaranteed or endorsed by the publisher.
